# Metabolic (Dysfunction)-Associated Fatty Liver Disease in Chinese Patients with Type 2 Diabetes from a Subcenter of the National Metabolic Management Center

**DOI:** 10.1155/2022/8429847

**Published:** 2022-01-28

**Authors:** Conghui Guan, Songbo Fu, Donghu Zhen, Kuan Yang, Jinyang An, Yapei Wang, Chengxu Ma, Na Jiang, Nan Zhao, Jinjin Liu, Fang Yang, Xulei Tang

**Affiliations:** ^1^Department of Endocrinology, The First Hospital of Lanzhou University, Lanzhou, China; ^2^The First Clinical Medical College, Lanzhou University, Lanzhou, China

## Abstract

**Background:**

Few studies have investigated the epidemiological metabolic (dysfunction) associated with fatty liver disease (MAFLD) in China, especially among those with type 2 diabetes.

**Methods:**

We recruited 3553 patients aged 18-75 years with type 2 diabetes who underwent abdominal ultrasound and serum biochemical analyses. Patient information including demographic and anthropometric parameters was also collected.

**Results:**

Overall, 63.2% of type 2 diabetic patients had MAFLD. Among the MAFLD patients, the proportions of lean, nonobese, and obese MAFLD were 23.1%, 75.7%, and 24.3%, respectively, and the percentage of previously undiagnosed MAFLD was 42.2%. MAFLD patients were younger, had shorter diabetic duration, and had greater BMI, aspartate aminotransferase (AST), alanine aminotransferase (ALT), fasting insulin, postprandial insulin, total cholesterol, and insulin resistance levels (HOMA-IR and TyG index). Liver fibrosis diagnostic panels revealed that the proportions of elevated AST (≥40 U/L) and ALT (≥40 U/L) were 7.3% and 18.5%, respectively. The distributions of AST-to-platelet ratio index (APRI), fibrosis-4 (FIB-4) index, and nonalcoholic fatty liver disease fibrosis score (NFS) per stage were as follows: APRI—low 55.1%, indeterminate 35.3%, and high 9.5%; FIB-4—low 48.2%, indeterminate 45.3%, and high 6.5%; and NFS—low 15.0%, indeterminate 70.0%, and high 13.0%.

**Conclusions:**

MAFLD is a very common condition and generally had greater frequency of metabolic characteristics among type 2 diabetics in China. Many MAFLD patients were in the “indeterminate” or “high” stage when APRI, FIB-4, and NFS were assessed. Assessment of MAFLD should be included in the management of type 2 diabetes.

## 1. Introduction

Nonalcoholic fatty liver disease (NAFLD) is classically characterized by hepatic steatosis without any other etiological agents of excess fat accumulation in the liver (e.g., excessive drinking and viral hepatitis). It has become an increasingly frequent factor of end-stage liver complications and cardiovascular disease owing to its high prevalence (25.2% in global adult population) [1] in recent decades, posing a major impact on public health and a significant economic burden [2].

Of concern, the main feature of NAFLD is recognized to be a component of metabolic syndrome, which is also seen as a coexisting disease and primary driver in clinical practice [2]. Last year, a consensus statement given out by an international expert panel proposed that metabolic (dysfunction)-associated fatty liver disease (MAFLD) be used as the accurate terminology instead of NAFLD to reflect fatty liver associated with cardiometabolic factors following a set of simple, comprehensive, and easily applicable “positive” diagnoses independent of alcohol consumption [3, 4]. The newly proposed definition for MAFLD is based on hepatic steatosis along with the existence of one of the following three criteria: clinical evidence of metabolic dysfunction, type 2 diabetes, or overweight/obesity [4]. The Asian Pacific Association for the Study of the Liver (APASL) and the Chinese Society of Hepatology subsequently accepted the consensus to use MAFLD as a descriptor for fatty liver concomitant to metabolic dysfunction [5, 6]. Among such metabolic dysfunction comorbidities, type 2 diabetes is closely correlated with MAFLD and is almost the primary risk factor [7]. Thus, consistent with the growing prevalence of type 2 diabetes worldwide, the global prevalence and hazard of MAFLD have also been increasing rapidly [8]. A meta-analysis study reported the rate of type 2 diabetes as 22.51% among NAFLD patients and 43.63% among subjects with histologically proven nonalcoholic steatohepatitis (NASH) [1]. NAFLD was reported in 55.5% of people with type 2 diabetes with a >2-fold higher prevalence than in people without diabetes [9]. MAFLD is related with liver-related complications and adverse cardiometabolic outcomes [2], which support the bidirectional relationship with type 2 diabetes.

Despite its growing prevalence and concern [4], MAFLD is not properly evaluated as a familiar chronic hepatic disease, with only a few epidemiological investigations in Asia with no national surveys from any single country including China [5, 6]. Whether the expected increase in the prevalence rate of diabetes and obesity in China [10, 11] would cause a tremendous increase in the disease burden of MAFLD is a salient question. Accordingly, we aimed to use the information obtained from the National Metabolic Management Center (MMC) in Gansu province (Northwest China), including abdominal ultrasound, demographic parameters, liver chemistry, and diabetes testing profile for subjects with type 2 diabetes, to investigate the prevalence, characteristics, risk factors, and distributions of liver fibrosis scores of MAFLD. We expected this study to provide useful information on MAFLD and increase awareness regarding MAFLD in type 2 diabetic patients.

## 2. Materials and Methods

### 2.1. Participants

The MMC was established in 2016 to impart efficient diagnosis, treatment, comprehensive diabetes management, and long-term follow-up in China [12]. The First Hospital of Lanzhou University (Gansu, China) was one of the first hospitals involved in the MMC network and provided care to more than 4830 diabetes patients in Gansu province from February 2017 to May 2021. We excluded 275 participants who refused abdominal ultrasound and those with missing important covariates (e.g., liver chemistry, blood glucose, and insulin or demographic parameters), leaving 3553 participants with type 2 diabetes to be analyzed in the present study. All participants signed the written informed consent form, and the protocol of MMC has been approved by the Institutional Review Board of Ruijin Hospital (ClinicalTrials.gov number NCT03811470). All procedures were conducted in accordance with the Declaration of Helsinki.

### 2.2. Demographic and Anthropometric Parameters

An advanced online and offline proprietary medical record system for the MMC [12] was conducted to integrate demographic and anthropometric information (e.g., sex, age, diseases and medication history, lifestyle and dietary habits, height, and weight). Smoking was defined as “yes” if the patients smoked daily or almost daily. Drinking was defined as “yes” if the patients drank weekly or almost weekly, overlooking alcohol consumption. The consumption of vegetables per day was divided into <200 g, 200–400 g, 400–600 g, and ≥600 g. Body mass index (BMI) was counted as weight (kg)/height (m)^2^ and stratified as normal weight (<24.0 kg/m^2^), overweight (24.0-27.9 kg/m^2^), and obese (≥28.0 kg/m^2^), as per the guidelines for Chinese adults [13].

### 2.3. Laboratory Assays

Blood samples were obtained from participants after overnight fasting (at least 8–12 h). Postprandial blood samples were obtained 2 h after consumption of a steamed bread meal to measure postprandial glucose and postprandial insulin levels. Platelets (PLTs) were determined by flow cytometry, and aspartate aminotransferase (AST), alanine aminotransferase (ALT), albumin (ALB), plasma glucose, total cholesterol (TC), triacylglycerols (TG), HDL, and LDL were detected by an automatic biochemical analyzer in the Clinical Lab in our hospital. Serum insulin was determined by electrochemiluminescence immunoassay using immunoassay analyzers. Glycated hemoglobin (HbA_1c_) levels were measured using high-performance liquid chromatography. The homeostasis model assessment for insulin resistance (HOMA-IR) was counted as [fasting insulin (*μ*IU/mL) × fasting glucose (mmol/L)]/22.5, and triglyceride-glucose (TyG) was calculated as ln[fasting triglycerides (mg/dL) × fasting glucose (mg/dL)/2].

### 2.4. Definitions of Type 2 Diabetes and MAFLD

The 1999 World Health Organization diagnostic criteria were used to diagnose type 2 diabetes: fasting glucose ≥ 7.0 mmol/L, postprandial blood glucose ≥ 11.1 mmol/L, or self-reported diagnosed diabetes [14]. All participants underwent abdominal ultrasound examination (Madison Ultrasound Diagnostic Instrument SO-NoaCEX 8, Samsung, Korea) conducted by a senior sonographer in the Department of Ultrasound in our hospital. The imaging diagnosis of fatty liver needs to satisfy the following ultrasound findings: high echo in the proximal diffusing point of the liver, higher echo intensity in the liver than in the kidney, and unclear intrahepatic tube structure. The diagnosis of MAFLD was based on the ultrasonically diagnosed fatty liver and the presence of type 2 diabetes [4, 5].

### 2.5. Liver Fibrosis Scores

For the assessment of significant liver fibrosis, AST/ALT, AST-to-platelet ratio index (APRI), fibrosis-4 (FIB-4) index, and NAFLD fibrosis score (NFS) were determined according to published formulas as diagnostic panels. AST/ALT was defined as AST(U/L)/ALT(U/L) [15]; APRI as AST (U/L)/PLT (10^9^/L) × 100 [16]; FIB-4 as age (years) × AST (U/L)/[PLT (10^9^/L) × ALT (U/L)]^1/2^ [17]; and NFS as −1.675 + 0.037 × age (years) + 0.094 × BMI (kg/m^2^) + 1.13 + 0.99 × AST/ALT ratio − 0.013 × PLT (10^9^/L) − 0.66 × ALB (g/dL) [18]. For excluding advanced liver fibrosis, cutoff points were used as per the published studies. MAFLD patients are stratified into possessing low, intermediate, or high hazard for advanced fibrosis according to the following points: APRI (0.25 and 0.5), FIB-4 (1.30 and 2.67), and NFS (−1.455 and 0.676).

### 2.6. Statistical Analysis

**Figure 1:**
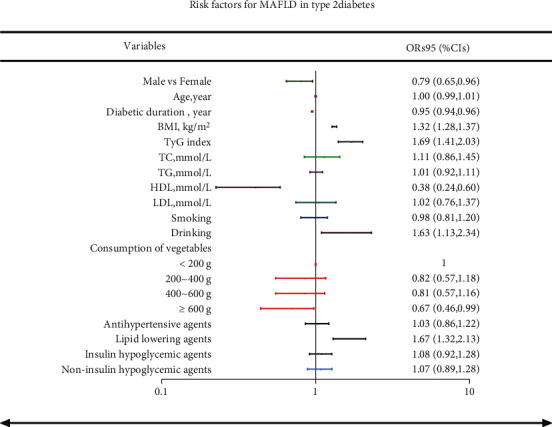
Risk factors for MAFLD in type 2 diabetes. Data are expressed as ORs (95%CIs). MAFLD: metabolic (dysfunction)-associated fatty liver disease; ORs: odds ratios; TyG: triglyceride-glucose index; TC: total cholesterol; TG: triacylglycerols; HDL: high-density lipoprotein cholesterol; LDL: low-density lipoprotein cholesterol.

The clinical characteristics of all subjects (Table 1) and patients with MAFLD according to BMI category (Table 2) are described. Data are shown as mean ± SD or median (25^th^ percentile, 75^th^ percentile) values for continuous variables and as frequency (%) for categorical variables. *P* values were calculated using *t-*tests, nonparametric tests, and chi-squared tests for normal distribution variables, nonnormal distribution variables, and classified variables. To identify the most important factors predicting the outcome of MAFLD in type 2 diabetes, a logistic regression model was performed (Figure 1). Confounders included sex, age, duration of diabetes, BMI, TyG index, lifestyle behaviors (smoking and drinking status), dietary habits (consumption of vegetables), and the use of antihypertensive agents, lipid-lowering agents, insulin, or noninsulin hypoglycemic agents. The results from the logistic regression model were presented as odds ratios (ORs) and 95% CIs. Statistical significance was defined as a two-sided *P* value < 0.05. The IBM SPSS Statistics 26 software (IBM Corporation, Armonk, NY, USA) was used for all analyses.

## 3. Results

### 3.1. Clinical Characteristics

The study included 3553 subjects aged 18-75 years with type 2 diabetes. Of the total patients, 66.3% were males; the mean age of the total study population was 56.1 (SD, 10.1) years, and the mean BMI was 25.3 (SD, 3.3) kg/m^2^. Among participants, 40.4% had a history of hypertension and 37.9% had a history of dyslipidemia. The prevalence of MAFLD was 63.2%. Among the MAFLD patients, the percentage of previously undiagnosed MAFLD was 42.2% (data not shown). Patients with MAFLD were younger, had a shorter diabetic duration and greater BMI, AST, ALT, ALB, fasting insulin, postprandial insulin, TC, TG, LDL, HOMA-IR, and TyG index levels, and included higher percentages of individuals with histories of smoking, drinking, hypertension, and dyslipidemia than non-MAFLD patients (all *P* < 0.05) (Table 1). The same study characteristics in Table 1, stratified by BMI, in patients with MAFLD are shown in Table 2. The proportions of lean (BMI < 24.0 kg/m^2^), nonobese (BMI < 28.0 kg/m^2^), and obese (BMI ≥ 28.0 kg/m^2^) MAFLD patients were 23.1%, 75.7%, and 24.3%, respectively. Obese MAFLD patients were younger, had lower levels of postprandial glucose and shorter diabetic duration, had greater levels of AST, ALT, fasting insulin, postprandial insulin, TG, HOMA-IR index, and TyG index, and had higher percentages of individuals with hypertension and dyslipidemia histories than subjects with lean or nonobese MAFLD (all *P* < 0.05). No significant associations were found in PLT, fasting glucose, TC, LDL, HbA_1c_, and the frequency of individuals with histories of smoking and drinking among lean, nonobese, and obese MAFLD (all *P* ≥ 0.05) (Table 2).

### 3.2. Risk Factors

A binary logistic regression analysis revealed that BMI, TyG index, drinking, and lipid-lowering agents were positively related to MAFLD presence (all *P* < 0.05), whereas male sex and diabetic duration were negatively related to MAFLD presence (all *P* < 0.05). Notably, among type 2 diabetics, those with ≥600 g consumption of vegetables had more than a 30% reduction in MAFLD risk compared to those with <200 g consumption (*P* < 0.05) (Figure 1).

### 3.3. Distributions of Liver Fibrosis Scores

**Figure 2:**
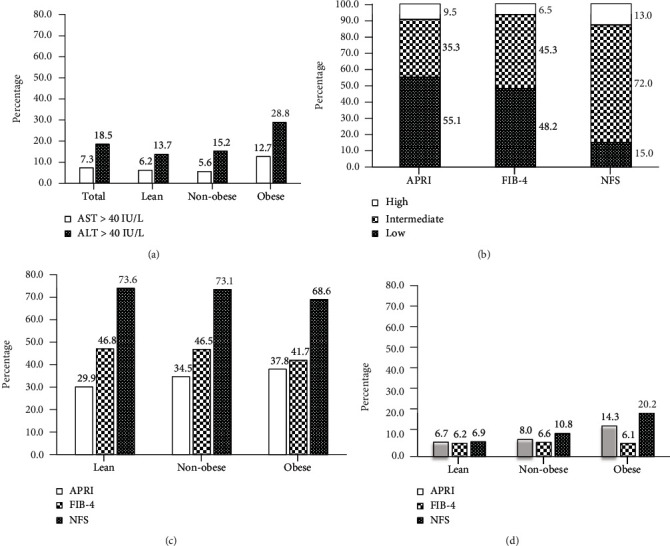
(a) Proportions of patients having plasma AST or ALT levels ≥ 40 U/L in total MAFLD, lean, nonobese, and obese MAFLD. (b) Distributions of noninvasive liver fibrosis scores in MAFLD patients. (c) Proportions of patients having “intermediate” risk of fibrosis for each score in lean, nonobese, and obese MAFLD patients. (d) Proportions of patients having “high” risk of fibrosis for each score in lean, nonobese, and obese MAFLD patients. AST: aspartic acid aminotransferase; ALT: alanine aminotransferase; APRI: AST-to-platelet ratio index; FIB-4: fibrosis-4 index; NFS: nonalcoholic fatty liver disease fibrosis score.

We also analyzed the distributions and levels of noninvasive liver fibrosis diagnostic panels (including AST/ALT, APRI, Fib-4, and NFS index) in lean, nonobese, and obese MAFLD patients (Figure 2). The entire distribution of liver fibrosis diagnostic scores and the statistic difference is shown in Supplementary Table 1. A significant correlation between BMI and liver fibrosis diagnostic scores was observed. Obese MAFLD patients had higher proportions of elevated AST, ALT, APRI, and NFS scores and a greater proportion of “high risk” for advanced fibrosis for APRI and NFS (APRI ≥ 0.5 and NFS ≥ 0.676).

## 4. Discussion

### 4.1. Prevalence and Awareness of MAFLD

Mainland China has seen a rapid increase in the burden of metabolic diseases owing to rapid economic transition and unhealthy lifestyles [19]. The latest prevalence of diabetes diagnosed by the American Diabetes Association criteria was 12.8% [11], and the rate of overweight/obesity exceeded 50% [10] among Chinese adults. MAFLD, earlier termed as NAFLD, was suggested as a more appropriate overarching nomenclature to emphasize the dominant driver of cardiometabolic elements in the process of fatty liver [3, 4]. This disease has affected more than one billion people worldwide [20], placing a major burden on the healthcare field and the economy.

This large single-center survey indicated that 63.2% of 18–75-year-old type 2 diabetics had MAFLD in China. This article is the first to focus on NAFLD/MAFLD in Chinese diabetes patients with a fair-sized sample volume and the first in China aimed at establishing the disease burden in type 2 diabetes patients seeking hospitalized care. Using the new MAFLD diagnostic criteria, one study from Hong Kong census database including 922 adults reported the prevalence of MAFLD as 25.9% [21]. Two studies, each including 384 and 339 patients with type 2 diabetes from Anhui province (East China), reported the prevalence of NAFLD as evaluated by ultrasonography to be 58.67% [22] and 68% [23], respectively. The 63.2% prevalence identified in our study is in line with these two studies in China and is higher than a global meta-analysis study based on ultrasound (which reported a prevalence of 55.5%) [9]. The prevalence from our study is lower than that observed by other investigators using transient elastography in European (Italy) [24] and Asian (Malaysia [25], Singapore [26], and Vietnam [27]) cohorts, as well as in the USA [28, 29], and is also lower than that using liver biopsy [30]. While MAFLD rates vary [4], the deviation between the present participants and the other Chinese studies may be ascribed to the special patients from Gansu province (an economically underdeveloped region in China) and the modest sensitivity of ultrasound in our analysis. China has released clear national plans for viral hepatitis; however, policies on fatty liver disease are less well formulated than those for other liver diseases and metabolic comorbidities, such as cardiovascular disease and diabetes. MAFLD is usually overlooked in clinical practice, especially by doctors treating diabetes, although it affects more than half of type 2 diabetics. Significant fibrosis was associated with increased risk of macrovascular and microvascular complications in type 2 diabetes; moreover, no recommendations have been included in the guidelines for the prevention and treatment of type 2 diabetes mellitus in China (2020 edition) [31] to date. In this survey, over 40% of MAFLD were previously undiagnosed patients, suggesting that the healthcare system needs further attention and more screening methods for MAFLD in the diabetes division. Type 2 diabetic Chinese patients with MAFLD were more youthful and had shorter diabetic duration than non-MAFLD patients, which encouraged us to regularly detect hepatic steatosis in newly diagnosed diabetes. Meanwhile, diet, lifestyle changes, and antidiabetic medications [32] may be beneficial for MAFLD.

### 4.2. Metabolic Characteristics

MAFLD is considered a metabolic hepatic disease. Recognizing the characteristics of MAFLD is instrumental in developing guidance for patient care. This survey indicated that MAFLD patients had greater BMI, lipid, and insulin resistance (e.g., high fasting and postprandial insulin, HOMA-IR, and TyG index) levels and had higher proportions of those who smoked and drank than those without MAFLD. However, the binary logistic regression suggested that age, TC, TG, LDL, smoking, and hypoglycemic agents had no relationship with the presence of MAFLD. In most metabolic studies, Asians were more likely to get insulin resistance than Caucasians [33] or European individuals [34] despite having a parallel or lower BMI. Therefore, specific BMI cutoff points have been proposed for the Asian people [35], particularly the Chinese [13]. The risk causes for MAFLD in Asians and those in Westerners are coincidental [5]. The role of dietary habits, such as the amount of vegetables consumed, has not been well assessed. Notably, in type 2 diabetics, those with ≥600 g consumption of vegetables had more than a 30% reduction in MAFLD risk than those with <200 g consumption. Although the incidence of MAFLD is notably increased accompanied by BMI, it is also being diagnosed increasingly in lean and nonobese persons. The proportions of lean (23.1%) and nonobese MAFLD (75.7%) patients in the type 2 diabetes population were higher than those of MAFLD patients in the general crowd (lean: 19.2%, nonobese: 40.8%) [36]. Unlike MAFLD versus non-MAFLD, there were no significant associations between fasting glucose, TC, LDL, HbA_1c_, and the frequency of individuals who smoked and drank when comparing obese versus nonobese or lean MAFLD patients. These outcomes indicate that obesity has more effect on liver enzymes and insulin resistance than glucose and lipids for MAFLD. Thus, adopting the proposed new definition of fatty liver (MAFLD) has multiple advantages, including capturing its primary drivers, the entire spectrum of the disease, and coexisting disease modifiers in order to promote improved lifestyles on one's own initiative and have positive public health and political outcomes. No approved drug therapy currently exists [4], and lifestyle modifications containing diet and exercise are still cornerstone intervention means [5]. Lessons can be drawn from diabetologists and cardiologists who have successfully treated diseases by correcting unhealthy lifestyle, obesity, smoking, and alcohol abuse.

### 4.3. Noninvasive Tests (Liver Fibrosis Scores)

The majority of MAFLD patients are stable and asymptomatic until they progress to inflammatory injury or cirrhosis [2]. One of the strongest causes in the development of MAFLD to cirrhosis or other end-stage liver disease is type 2 diabetes [4]. A meta-analyses found that fibrosis progression accelerated significantly more in NASH than in nonalcoholic fatty liver (NAFL, the early stage of MAFLD) [37] and that nearly 20% of NASH patients can be sorted into “rapid progressors” [38]. To distinguish the “rapid progressors” is an urgent issue, especially for diabetes. The International Expert Consensus Statement [4] and ASPAL [5] both recommended that ultrasound is the preferred checking method for hepatic steatosis. Noninvasive fibrosis diagnostic scores, such as APRI, FIB-4, and NFS, are commonly used to exclude advanced fibrosis because of their fine negative predictive values, in spite of the modest accuracy [5]. Although transient elastography is more precise than ultrasound and is widely used in liver disease branch [39], it is also expensive and still cannot replace liver biopsy. ASPAL suggested that transient elastography and/or fibrosis diagnostic scores are inferior to liver biopsy for confirming significant or advanced fibrosis and require further confirmation [5]. When biochemical diagnostic scores in type 2 diabetics with MAFLD were assessed in our study, the proportions of patients in the “indeterminate” and “high” zones were 44.9~85.0%. Whether they all require liver biopsy is a pertinent question. Chan et al. [40] indicated that the use of NFS or FIB-4 together with transient elastography for patients with intermediate or high scores appeared to be the optimal approach. A standardized official diagnostic method and procedure for MAFLD should be considered.

### 4.4. Limitations

The strength of this study is incorporating plentiful patients diagnosed with type 2 diabetes who underwent standardized abdominal ultrasonography and laboratory measurement. Our study may still have some limitations. First, this single-center design failed to investigate the comprehensive condition of MAFLD in type 2 diabetes Chinese patients. Second, for the detection of steatosis, ultrasound has limited sensitivity for light fatty liver and in individuals with a BMI > 40 kg/m^2^ [4]. However, ultrasonography scan is still proposed as the preferred means for MAFLD [4, 5]. Finally, the hospitalized type 2 diabetic participants may limit the universality of the findings. Yet, despite these deficiencies, this research is the first to demonstrate the condition of MAFLD in Chinese patients with type 2 diabetes to a great extent. These findings may provide useful information to guide policymaking in the course of MAFLD management in type 2 diabetics.

### 4.5. Conclusions and Policy Implications

MAFLD is a very common condition and generally had greater frequency of metabolic characteristics among type 2 diabetics in China. Many MAFLD patients were classified in the “intermediate” or “high” stage after APRI, FIB-4, and NFS assessment. This research reminded doctor, drug companies, and administrative departments to emphasize the need for better treatment regimens and attach the assessment of MAFLD and related liver fibrosis into the management procedure of type 2 diabetes.

## Figures and Tables

**Table 1:** Clinical characteristics of the study population.

Clinical characteristics	Non-MAFLD	MAFLD	Total
Participants	1306 (36.8)	2247 (63.2)	3553 (100.0)
Male	859 (65.8)	1497 (66.6)	2356 (66.3)
Age (years)	57.5 ± 9.7	55.4 ± 10.2^∗^	56.1 ± 10.1
Diabetes duration (years)	9.0 (3.1, 14.2)	5.2 (0.6, 10.6)^∗^	6.3 (1.2, 12.1)
BMI (kg/m^2^)	23.7 ± 2.8	26.2 ± 3.1^∗^	25.3 ± 3.3
Normal weight (<24.0 kg/m^2^)	753 (57.7)	519 (23.1)	1272 (35.8)
Overweight (24.0-27.9 kg/m^2^)	471 (36.1)	1183 (52.6)^∗^	1654 (46.6)
Obesity (≥28.0 kg/m^2^)	82 (6.3)	545 (24.3)^∗^	627 (17.6)
PLT (10^9^/L)	183.0 ± 60.6	186.8 ± 54.8	185.4 ± 57.0
AST (U/L)	19 (16, 24)	21 (17, 21)^∗^	20 (16, 26)
ALT (U/L)	18 (13, 26)	24 (17, 35)^∗^	21 (15, 32)
ALB (g/dL)	4.21 ± 0.40	4.37 ± 0.36^∗^	4.31 ± 0.38
Fasting glucose (mmol/L)	9.53 ± 3.86	9.41 ± 3.10	9.45 ± 3.40
Fasting insulin (*μ*IU/mL)	6.09 (3.83, 8.84)	8.35 (5.81, 11.72)^∗^	7.48 (4.97, 10.87)
Postprandial glucose (mmol/L)	17.62 ± 6.12	17.19 ± 5.37^∗^	17.35 ± 5.66
Postprandial insulin (*μ*IU/mL)	20.56 (12.03, 34.49)	28.09 (17.59, 45.91)^∗^	25.22 (15.45, 42.03)
TC (mmol/L)	4.30 ± 1.04	4.51 ± 1.14^∗^	4.43 ± 1.11
TG (mmol/L)	1.27 (0.93, 1.78)	1.82 (1.32, 2.69)^∗^	1.60 (1.13, 2.37)
HDL (mmol/L)	1.10 ± 0.27	1.01 ± 0.25^∗^	1.05 ± 0.26
LDL (mmol/L)	2.76 ± 0.80	2.92 ± 0.81^∗^	2.86 ± 0.81
HbA_1c_ (%)	8.58 ± 2.25	8.67 ± 2.03	8.63 ± 2.11
HOMA-IR	2.26 (1.42, 3.63)	3.25 (2.14, 4.93)^∗^	2.88 (1.81, 4.52)
TyG index	9.15 ± 0.70	9.55 ± 0.73^∗^	9.40 ± 0.75
Smoking	348 (26.6)	672 (29.9)^∗^	1020 (28.7)
Drinking	54 (4.1)	168 (7.5)^∗^	222 (6.2)
Hypertension history	496 (38.0)	938 (41.7)^∗^	1434 (40.4)
Dyslipidemia history	308 (23.6)	1040 (46.3)^∗^	1348 (37.9)

Data are expressed as mean ± SD, median (25th percentile, 75th percentile), or *n* (%). MAFLD: metabolic (dysfunction)-associated fatty liver disease; BMI: body mass index; PLT: platelet; AST: aspartic acid aminotransferase; ALT: alanine aminotransferase; ALB: albumin; TC: total cholesterol; TG: triacylglycerols; HDL: high-density lipoprotein cholesterol; LDL: low-density lipoprotein cholesterol; HbA_1c_: glycated hemoglobin; HOMA-IR: homeostasis model assessment for insulin resistance; TyG: triglyceride-glucose index. ^∗^*P* < 0.05 for comparisons of MAFLD versus non-MAFLD.

**Table 2:** Clinical characteristics in different MAFLD categories stratified by BMI.

Clinical characteristics	MAFLD (*n* = 2247)		
	Lean (BMI < 24.0 kg/m^2^)	Nonobese (BMI < 28.0 kg/m^2^)	Obese (BMI ≥ 28.0 kg/m^2^)
Participants	519 (23.1)	1702 (75.7)	545 (24.3)
Male	298 (57.4)^∗^	1102 (64.7)^†^	395 (72.5)
Age (years)	56.4 ± 8.8^∗^	56.0 ± 9.7^†^	53.3 ± 11.3
Diabetes duration (years)	5.3 (0.8, 10.7)^∗^	5.6 (0.9, 11.0)^†^	3.3 (0.1, 9.2)
BMI (kg/m^2^)	22.5 ± 1.3^∗^	24.9 ± 1.9^†^	30.3 ± 2.5
PLT (10^9^/L)	189.7 ± 53.9	186.1 ± 54.2	188.8 ± 56.6
AST (U/L)	19 (16, 24)^∗^	20 (16, 26)^†^	23 (18, 31)
ALT (U/L)	20 (15, 29)^∗^	22 (16, 32)^†^	29 (20, 43)
ALB (g/dL)	4.33 ± 0.34	4.37 ± 0.36	4.36 ± 0.33
Fasting glucose (mmol/L)	9.41 ± 3.13	9.41 ± 3.07	9.39 ± 3.20
Fasting insulin (*μ*IU/mL)	6.70 (4.41, 9.56)^∗^	7.77 (5.41, 10.72)^†^	10.80 (7.49, 14.51)
Postprandial glucose (mmol/L)	17.93 ± 5.63^∗^	17.43 ± 5.41^†^	16.44 ± 5.17
Postprandial insulin (*μ*IU/mL)	23.89 (14.92, 40.73)^∗^	26.85 (16.96, 43.32)^†^	33.26 (19.94, 55.58)
TC (mmol/L)	4.56 ± 1.04	4.51 ± 1.14	4.51 ± 1.14
TG (mmol/L)	1.73 (1.23, 2.57)^∗^	1.79 (1.30, 2.61)^†^	1.96 (1.43, 3.05)
HDL (mmol/L)	1.05 ± 0.25^∗^	1.02 ± 0.23	1.00 ± 0.30
LDL (mmol/L)	2.95 ± 0.80	2.92 ± 0.82	2.92 ± 0.77
HbA_1c_ (%)	8.79 ± 2.17	8.67 ± 2.04	8.66 ± 2.00
HOMA-IR	2.51 (1.69, 4.00)^∗^	3.00 (2.01, 4.48)^†^	4.30 (2.86, 6.08)
TyG index	9.47 ± 0.76^∗^	9.52 ± 0.74^†^	9.63 ± 0.72
Smoking	221 (27.8)	569 (28.8)	167 (30.6)
Drinking	51 (6.4)	143 (7.2)	38 (7.0)
Hypertension history	167 (32.2)^∗^	665 (39.1)^†^	273 (50.1)
Dyslipidemia history	207 (39.9)^∗^	753 (44.2)^†^	287 (52.7)

Data are expressed as mean ± SD, median (25th percentile, 75th percentile), or *n* (%). MAFLD: metabolic (dysfunction)-associated fatty liver disease; BMI: body mass index; PLT: platelet; AST: aspartic acid aminotransferase; ALT: alanine aminotransferase; ALB: albumin; TC: total cholesterol; TG: triacylglycerols; HDL-C: high-density lipoprotein cholesterol; LDL-C: low-density lipoprotein cholesterol; HbA_1c_: glycated hemoglobin; HOMA-IR: homeostasis model assessment for insulin resistance; TyG: triglyceride-glucose index. ∗ and † indicated *P* < 0.05 for comparisons lean versus obese and nonobese versus obese MAFLD.

## Data Availability

All data used to support the findings of this study are available from the corresponding author upon reasonable request.

## Supplementary Materials

Supplementary Materials Supplementary Table 1: the entire distribution of liver fibrosis diagnostic scores and the statistic difference according to different MAFLD categories.
