# Supplementary material for: Metabolic (Dysfunction)-Associated Fatty Liver Disease in Chinese Patients with Type 2 Diabetes from a Subcenter of the National Metabolic Management Center
Source: J Diabetes Res. 2022 Jan 28;2022:8429847. doi: 10.1155/2022/8429847 (PMC8816602; doi:10.1155/2022/8429847)
Supplement: Supplementary Materials — Supplementary Table 1: the entire distribution of liver fibrosis diagnostic scores and the statistic difference according to different MAFLD categories. [file 8429847.f1.docx]

Supplemental Table 1 Liver fibrosis diagnostic panel according to different MAFLD categories

| Clinical Characteristics | MAFLD | | | |
| --- | --- | --- | --- | --- |
|  | Total | Lean- MAFLD | Non-obese-MAFLD | Obese- MAFLD |
| AST/ALT | 0.86(0.71,1.06) | 0.93(0.77,1.15)﹡ | 0.88(0.73,1.08) † | 0.80(0.66,0.95) |
| AST>40 U/L | 165(7.3) | 32(6.2)﹡ | 96(5.6) † | 69(12.7) |
| ALT>40 U/L | 416(18.5) | 71(13.7)﹡ | 259(15.2) † | 157(28.8) |
| APRI | 0.24(0.17,0.33) | 0.21(0.16,0.30)﹡ | 0.23(0.17,0.32) † | 0.26(0.18,0.39) |
| Low (<0.25) | 1239(55.1) | 329(63.4) | 978(57.5) | 261(47.9) |
| Intermediate (0.25~0.5) | 794(35.3) | 155(29.9) | 588(34.5) | 206(37.8) |
| High (≥0.5) | 214(9.5) | 35(6.7) | 136(8.0) | 78(14.3) |
|  |  | ***p*<0.001** | ***p*<0.001** |  |
| FIB-4 | 1.33(0.98,1.78) | 1.33(1.01,1.75) | 1.34(0.99,1.78) | 1.27(0.92,1.74) |
| Low (<1.30) | 1084(48.2) | 244(47.0) | 799(46.9) | 285(52.3) |
| Intermediate (1.30~2.67) | 1018(45.3) | 243(46.8) | 791(46.5) | 227(41.7) |
| High (≥2.67) | 145(6.5) | 32(6.2) | 112(6.6) | 33(6.1) |
|  |  | *P*=0.212 | *P*=0.093 |  |
| NFS | -0.39(-1.07,0.23) | -0.64(-1.28,-0.02)﹡ | -0.45(-1.13,0.16) † | -0.21(-0.82,0.54) |
| Low (< -1.455) | 336(15.0) | 101(19.5) | 275(16.2) | 61(11.2) |
| Intermediate (-1.455~0.676) | 1618(72.0) | 382(73.6) | 1224(73.1) | 374(68.6) |
| High (≥0.676) | 293(13.0) | 36(6.9) | 183(10.8) | 110(20.2) |
|  |  | ***p*<0.001** | ***p*<0.001** |  |

Data are expressed as median (interquartile range) or n (%). MAFLD, metabolic (dysfunction) associated fatty liver disease; AST, aspartic acid aminotransferase; ALT, alanine aminotransferase; APRI, AST-to-platelet ratio index; FIB-4, Fibrosis-4 index; NFS, NAFLD fibrosis score. * and † indicated *P*<0.05 for comparisons lean- versus obese-, and non-obese- versus obese-MAFLD. *P* values were calculated by Chi-square categorical variables across the three groups.
